# TMT Based Proteomic Analysis of Human Follicular Fluid From Overweight/Obese and Normal-Weight Patients With Polycystic Ovary Syndrome

**DOI:** 10.3389/fendo.2019.00821

**Published:** 2019-11-26

**Authors:** Xinyi Zhang, Xiaoyan Xu, Pingping Li, Feifei Zhou, Lin Kong, Jiahui Qiu, Zhengwei Yuan, Jichun Tan

**Affiliations:** ^1^Reproductive Medical Center, Obstetrics and Gynecology Department, Shengjing Hospital of China Medical University, Shenyang, China; ^2^Key Laboratory of Reproductive Dysfunction Diseases and Fertility Remodeling of Liaoning Province, Shenyang, China; ^3^Key Laboratory of Health Ministry for Congenital Malformation, Shengjing Hospital of China Medical University, Shenyang, China

**Keywords:** polycystic ovary syndrome, follicular fluid, obesity, proteomics, tandem mass tag

## Abstract

**Background:** Polycystic ovary syndrome (PCOS) is a major endocrine and metabolic disorder with heterogeneous manifestations and complex etiology. As a leading cause of anovulatory infertility, the molecular diversity of the follicular microenvironment has not been fully elucidated. The aim of the present study was to investigate the follicular fluid proteomic profiles of overweight/obese and normal-weight women with PCOS, to identify novel molecular mechanisms underlying PCOS and to determine the effect of obesity on the follicular fluid protein profiles.

**Methods:** Follicular fluid samples were collected from 3 different groups: overweight/obese PCOS patients (*n* = 29), normal-weight PCOS patients (*n* = 29), and normo-ovulatory controls (*n* = 29). We used a quantitative approach with tandem mass tag labeling and liquid chromatography tandem mass spectrometry to identify the differentially expressed proteins. Differential abundance of four selected proteins was confirmed by ELISA. Gene Set Enrichment Analysis was also conducted to further explore our findings. Furthermore, we compared the clinical, hormonal, and biochemical characteristics of overweight/obese and normal-weight patients with PCOS to determine the effects of obesity.

**Results:** A total of 1,153 proteins were identified, of which 41 and 19 proteins were differentially expressed in the overweight/obese PCOS group vs. the control group, and in the normal-weight PCOS group vs. the control group, respectively. Bioinformatics analyses showed that the inflammatory, immunological, and metabolic-related biological processes were co-enriched in both subgroups of PCOS. Apolipoprotein A-II, complement C5, fetuin-B, and stromal cell-derived factor 1 were found to be involved in various processes and were validated using the ELISA analysis. From clinical features and proteomic data, obesity was found to worsen follicular development disturbances in PCOS.

**Conclusion:** In this proteomic study, a panel of proteins were found differentially expressed in the follicular fluid of PCOS. Inflammatory, immunological, and metabolic abnormalities were identified inside the intra-follicular environment, which could be aggravated by obesity. The identified proteins were correlated with follicular growth and may be considered as candidate biomarkers as well as therapeutic targets of PCOS.

## Introduction

Polycystic ovary syndrome (PCOS) is one of the most common endocrine and metabolic disorders with a prevalence ranging from 5 to 10% among women of reproductive age (1, 2). Clinical manifestations include menstrual irregularities, anovulatory infertility, signs of androgen excess, metabolic and psychological disorders (3). Owing to its highly heterogeneous features and complex pathogenesis of PCOS, the diagnosis and treatment strategies for PCOS remain a matter of debate. Hence, a better understanding of the molecular mechanisms underlying PCOS could benefit the identification of novel diagnostic and therapeutic targets.

As the leading cause of anovulatory infertility, anovulation is the main characteristic of PCOS. Follicular fluid (FF) serves as the complex microenvironment for oocyte growth, follicular maturation, and germ cell-somatic cell communications (4, 5). A series of immune cytokines have been identified differentially expressed in the FF of PCOS women (6, 7). Furthermore, cytokines, growth factors, proteins, metabolites, and microRNAs found in FF have been associated with oocyte competence, while angiotensin-(1–7) levels in human FF have been correlated with oocyte maturation (8, 9). Impaired oocyte quality and outcomes of *in-vitro* fertilization (IVF) in PCOS have been linked to changes in the components of FF, yet the mechanisms have not been fully elucidated (7, 10).

Women with PCOS are often overweight or obese (11, 12). Obesity has been reported to worsen hyperandrogenism, insulin resistance (IR), metabolic, and reproductive outcomes of PCOS patients (13, 14). Obese women with PCOS have lower clinical pregnancy and live birth rates than lean women with PCOS (15). Weight loss may improve endocrine disorders, the incidence of ovulation, responses to ovulation induction therapy, and pregnancy outcomes in obese PCOS women (16). Metabolic abnormalities remain throughout PCOS patients' lives and aging has been reported to influence IR through different mechanisms in obese and lean patients, indicating that PCOS is a syndrome comprised of body mass index (BMI)-determined sub-populations (17).

Proteomic analysis is an efficient approach for identifying novel disease patterns and for revealing effective diagnostic and therapeutic targets. Expression of proteins related to glucose and lipoprotein metabolism, IR, cell proliferation, and apoptosis have been reported through a two-dimensional gel electrophoresis analysis of FF from PCOS patients (18). Several proteins in serum samples have been identified as biomarkers that can predict the development of ovarian hyperstimulation syndrome in women with PCOS (19). Moreover, proteins involved in extracellular matrix remodeling, complement coagulation cascade, lipid transport, and angiogenesis have been found as dysregulated in the FF of PCOS (20). However, there have been no studies to date on whether excessive BMI would have a pathophysiological impact on FF protein profiles in PCOS. Hence, sub-group studies are needed among PCOS patients to reveal the role of obesity in PCOS.

In the present study, we conducted a tandem mass tag (TMT) based quantitative proteomic study to compare proteomes of FF from 3 groups: overweight/obese PCOS patients, normal-weight PCOS patients and healthy controls to further understand the molecular defects of PCOS. Furthermore, we compared the clinical, hormonal, and biochemical characteristics of overweight/obese and normal-weight PCOS patients to determine the effects of obesity on FF profiles in PCOS.

## Materials and Methods

### Study Subjects and Sample Collection

This study was approved by the Ethics Committee at Shengjing Hospital of China Medical University. Informed written consent was obtained from all of the participants. Eligible women (22–35 years of age) who had undergone IVF were recruited from July 2017 to July 2018. PCOS was diagnosed based on the Rotterdam criteria with two of three items: oligo- and/or anovulation, polycystic ovarian morphology on ultrasound, and hyperandrogenism (clinical or biochemical). PCOS patients were further divided into overweight/obese PCOS (Po) group with BMI ≥ 25 kg/m^2^ and normal-weight PCOS (Pn) group with BMI < 25 kg/m^2^. The control group included women who seek treatment for tubal infertility or male factors, with normal ovarian reserve (regular menstrual cycles, and AMH concentration of ≥1.1 ng/mL), and normal BMI. Women with endometriosis, cancer, or other medical disorders that could affect folliculogenesis were excluded. A total of 123 PCOS patients (75 Po patients and 48 Pn patients) were recruited. The clinical, hormonal, and biochemical characteristics were compared between the two subgroups.

FF samples were collected from 9 Po patients, 9 Pn patients, and 9 controls of the participants for proteomic analysis. Patients included met all the three items of Rotterdam criteria. FF samples from an additional 20 Po patients, 20 Pn patients, and 20 controls of the participants were collected for ELISA validation of the identified proteins. All subjects underwent controlled ovarian stimulation using the gonadotropin-releasing hormone antagonist protocol. FF was collected by transvaginal ultrasound-guided aspiration, 35–37 h after the administration of recombinant human chorionic gonadotropin (Ovidrel, Merck-Serono, Switzerland). Only clear FF samples with no macroscopic blood contamination were included. After oocyte isolation, the FF samples were centrifuged at 800 g for 10 min to remove cells and insoluble particles. The supernatant was then separated and stored at −80°C for further use.

### Depletion of Highly Abundant Proteins

FF samples were thawed and removed of any possible cellular debris by centrifugation at 12,000 g, at 4°C for 10 min. Pools of FF samples taken from three donors were prepared. Three separate pools of Po, Pn, and control samples were analyzed in parallel. The ProteoMiner Protein Enrichment Kit (BIO-RAD, California, USA) was used to remove highly abundant proteins. Protein concentrations after depletion were determined by bicinchoninic acid protein concentration assay (Beyotime Biotechnology, China) according to the manufacturer's instructions.

### Trypsin Digestion

Proteins were first reduced through exposure to 5 mM dithiothreitol at 56°C for 30 min and was subsequently alkylated using a solution of 11 mM iodoacetamide, for 15 min at room temperature in the dark. The sample was diluted with 100 mM tetraethylammonium tetrahydroborate (TEAB) to a concentration of urea of <2 M. Trypsin was added at a mass ratio of 1:50 (trypsin: protein) for the first digestion overnight, and a mass ratio of 1:100 (trypsin: protein) for a subsequent second digestion lasting for 4 h.

### TMT-Labeling and HPLC Fractionation

After trypsin digestion, the peptide was desalted using a Strata X C18 SPE column (Phenomenex, California, USA) and vacuum-dried. The peptide was solubilized with 0.5 M TEAB and labeled using the TMT kit (ThermoFisher, Massachusetts, USA) according to the manufacturer's instructions. Briefly, the thawed TMT reagent was reconstituted in acetonitrile. Peptide mixtures were incubated for 2 h at room temperature and pooled, desalted, and dried by vacuum centrifugation. Three Po samples were labeled with 127N, 127C, and 128N. Three Pn samples were labeled with 128C, 129N, and 129C. Three control samples were labeled with 130N, 130C, and 131.

The tryptic peptides were then fractionated by high pH reverse-phase HPLC using an Agilent 300Extend C18 column (250 mm in length, 5 μm particles, and 4.6 mm inner diameter). In brief, the peptides were separated with a gradient of 8 to 32% acetonitrile at pH 9.0 into 60 fractions for an hour. Then, the peptides were combined into 18 fractions and were vacuum freeze-dried until the subsequent operations.

### Liquid Chromatography Tandem Mass Spectrometry Analysis

Peptides dissolved in solvent A (aqueous solution with 0.1% formic acid and 2% acetonitrile) were loaded onto a reversed-phase analytical column. The gradient was set at an increase from 9 to 25% solvent B (aqueous solution with 0.1% formic acid and 90% acetonitrile) over 26 min, from 25 to 38% in 8 min, and climbing to 80% in 3 min, and then holding at 80% for 3 min, all at a constant flow rate of 700 nL/min on an EASY-nLC 1000 Ultra Performance Liquid Chromatography (UPLC) system (Thermo Scientific, Massachusetts, USA). The peptides were then subjected to a nanospray-ionization source followed by tandem mass spectrometry (MS/MS) in Orbitrap Fusion^TM^ (Thermo Scientific, Massachusetts, USA) coupled online to the UPLC. An electrospray voltage of 2.0 kV was applied. The m/z scan range was set 350 to 1,550 for a full scan and any intact peptides were detected on Orbitrap at a resolution of 60,000. The fixed first mass was set as 100 m/z. Peptides were then selected for MS/MS and the fragments were detected by Orbitrap at a resolution of 15,000. A data-dependent procedure alternated between one MS scan followed by 20 MS/MS scans with 30.0s dynamic exclusion. Automatic gain control was set at 5E4. The mass spectrometry proteomics data have been deposited to the ProteomeXchange Consortium (http://proteomecentral.proteomexchange.org) by the PRIDE partner repository with the dataset identifier PXD013937.

### Data Analysis and Database Search

The resulting MS/MS data were searched using the Maxquant software (v.1.5.2.8). Tandem mass spectra were searched using the SwissProt Human database concatenated with the reverse decoy database. Trypsin/P was specified as the cleavage enzyme allowing up to 2 missing cleavages. The mass tolerance for precursor ions was set as 20 ppm for the First search and as 5 ppm for the Main search. The mass tolerance of fragment ions was set as 0.02 Da. Carbamidomethyl residues on Cys were specified as the fixed modification and the oxidation on Met was specified as variable modifications. The false discovery rate (FDR) was adjusted to <1%, and the minimum score for peptides was set to >40. Differentially expressed protein (DEP) was identified as that with over 1.2- or <1/1.2-fold change as the cut-off value. Proteins with a *P* < 0.05 calculated by Student's *t*-test were included in the DEP list.

### Bioinformatics

Gene Ontology (GO) annotation of the identified proteins was derived from the UniProt-GOA database (www.ebi.ac.uk/GOA/). Proteins that were not annotated by UniProt-GOA database were annotated by InterProScan software using a protein sequence alignment method. Proteins were classified by GO annotations based on three categories: biological process, cellular component, and molecular function. WoLF PSORT software (https://wolfpsort.hgc.jp/) was used to predict the subcellular localization of the identified proteins. Enrichment analysis of the functional annotation was conducted by a two-tailed Fisher's Exact test. A *P* < 0.05 was considered as significant. The KEGG database (https://www.genome.jp/kegg/) was used to perform the enrichment analysis of pathways. Cluster analyses of the functional enrichment were performed. Categories that were enriched at least in one of the clusters with a *P* < 0.05 were selected. The filtered *P*-value matrix was transformed by the function x = –log10 (*P*-value) and was then z-transformed. The z scores were clustered by the one-way hierarchical clustering and visualized using R-package “gplots.” To understand the complex interactions between the DEPs and to assess the potential underlying pathways, GeneMANIA (https://genemania.org/) was applied.

Gene Set Enrichment Analysis (GSEA) (http://software.broadinstitute.org/gsea/) was performed to interpret protein expression data using predefined gene sets taken from the Molecular Signatures Database version 6.2. Fold change values were exported for all identified proteins and sequentially analyzed. To correlate a gene signature with predefined gene sets, significance was determined using a running-sum statistic for the whole gene set. The normalized enrichment score (NES) was used as the primary index to examine the enrichment results of gene sets. Gene sets with |NES|>1 and nominal *P* < 0.05 were considered significantly enriched. Leading edge analysis was performed with the TOP 10 most significantly enriched gene sets of the GO-Biological Processes. All the overlapping genes were retrieved, and STRING (https://www.string-db.org) was used to identify interactions. RCircos package was used for data visualization.

### ELISA Validation

The differential abundance of four selected proteins was validated by ELISA using FF samples of 20 patients each from the Po, Pn, and control groups. Concentrations of apolipoprotein A-II (APOA2) (Elabscience, Wuhan, China), complement C5 (C5) (CUSABIO, Wuhan, China), fetuin-B (FETUB) (Abcam, Cambridge, UK), and stromal cell-derived factor 1 (SDF-1/CXCL12) (R&D Systems, Minneapolis, USA) in the FF samples were measured using the commercial ELISA kits according to the manufacturer's instructions. The collected FF samples were diluted to 1:20, 1:100, and 1:500 for the APOA2, C5, and FETUB ELISA tests, respectively.

### Statistical Analysis

Statistical analyses were performed using SPSS software version 23.0 (IBM Software, New York, USA). The results are presented as mean ± standard deviation. Comparative analysis of the quantitative data was performed using the two-tailed Student's *t*-test between the Po and Pn groups and a *P* < 0.05 was considered statistically significant.

## Results

### Quantitative Mass Spectrometry Analysis of FF

**Figure 1:**
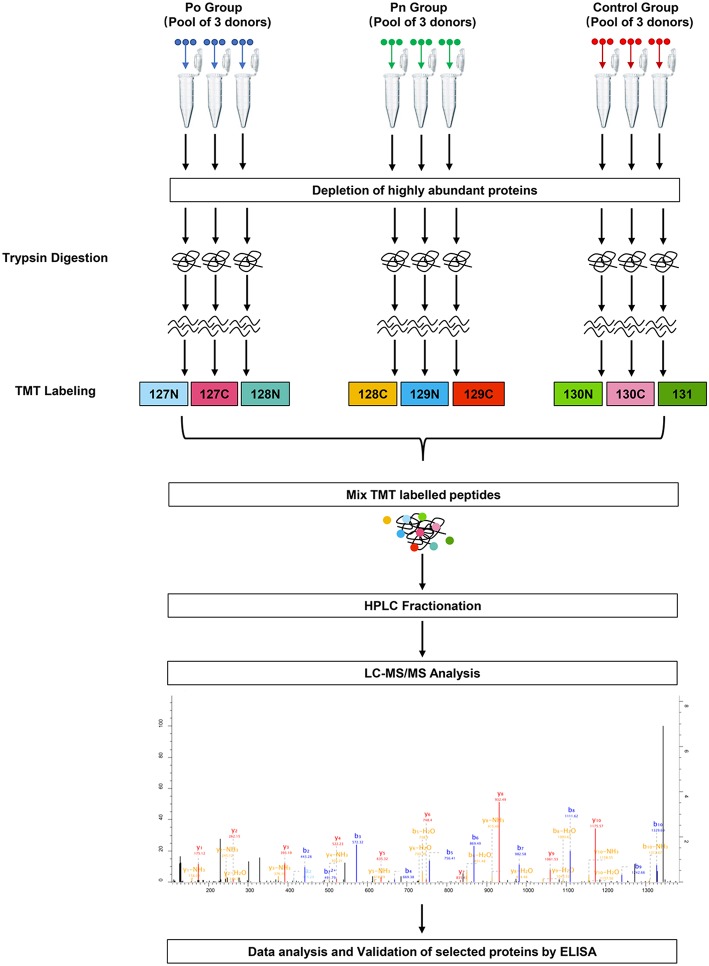
Schematic workflow of TMT based quantitative proteomic analyses of follicular fluid from overweight/obese PCOS patients, normal-weight PCOS patients and the controls.

**Figure 2:**
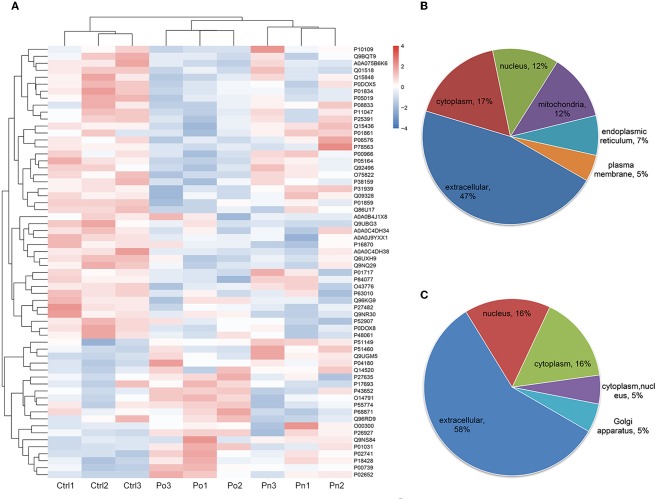
**(A)** Clustering heat map of the protein expression patterns of different groups. Red color indicates higher expression while blue indicates lower expression. Proteins without significantly differential expression were shown in white. **(B,C)** Distribution of the subcellular localization of differentially expressed proteins for Po vs. control and Pn vs. control.

To investigate the global proteome profiling of FF in the Po, Pn, and control groups, we performed the quantitative proteomic analysis on pooled FF samples (Figure 1). A total of 1,153 proteins were identified, of which 959 proteins were quantifiable. In total, 41 proteins were differentially expressed between Po vs. control groups (Po/C), and 19 were differentially expressed between Pn vs. control groups (Pn/C). The annotation and quantification information of DEPs are presented in Tables 1, 2. Three DEPs in common were found: upregulation of C-reactive protein (CRP), downregulation of immunoglobulin kappa constant (IGKC) and inactive serine protease PAMR1 (PAMR1). Changing amounts of DEPs from each of the pooled samples were illustrated using unsupervised hierarchical clustering heat map (Figure 2A). The subcellular localization of DEPs indicated that most proteins were localized extracellularly in both Po/C (47%) and Pn/C (58%) (Figures 2B,C).

**Table 1:** List of differentially expressed proteins identified in the follicular fluid from overweight/obese patients with PCOS compared with the controls.

**Accession**	**Description**	**Coverage %**	**Unique peptides**	**Fold change**	***P*-value**
P02741	C-reactive protein GN = CRP	24.6	7	4.933	0.025
P00739	Haptoglobin-related protein GN = HPR	33.3	2	1.520	0.027
P02652	Apolipoprotein A-II GN = APOA2	40.0	5	1.373	0.016
P01031	Complement C5 GN = C5	38.8	65	1.343	0.007
O14791	Apolipoprotein L1 GN = APOL1	30.9	17	1.318	0.013
O00300	Tumor necrosis factor receptor superfamily member 11B GN = TNFRSF11B	23.7	11	1.310	0.003
P43652	Afamin GN = AFM	41.4	27	1.284	0.006
P26927	Hepatocyte growth factor-like protein GN = MST1	48.0	26	1.249	0.006
P27635	60S ribosomal protein L10 GN = RPL10	14.0	3	1.206	0.048
Q86U17	Serpin A11 GN = SERPINA11	13.3	6	0.823	0.005
Q15436	Protein transport protein Sec23A GN = SEC23A	2.9	2	0.818	0.032
P11047	Laminin subunit gamma-1 GN = LAMC1	20.0	26	0.816	0.024
O43776	Asparagine–tRNA ligase, cytoplasmic GN = NARS	4.4	2	0.814	0.018
Q09328	Alpha-1,6-mannosylglycoprotein 6-beta-N-acetylglucosaminyltransferase A GN = MGAT5	3.8	3	0.813	0.041
P01859	Immunoglobulin heavy constant gamma 2 GN = IGHG2	50.0	7	0.801	0.030
P05164	Myeloperoxidase GN = MPO	20.5	12	0.793	0.008
P25391	Laminin subunit alpha-1 GN = LAMA1	11.3	31	0.789	0.045
P63010	AP-2 complex subunit beta GN = AP2B1	1.9	2	0.786	0.038
A0A0C4DH34	Immunoglobulin heavy variable 4-28 GN = IGHV4-28	7.7	1	0.784	0.050
O75822	Eukaryotic translation initiation factor 3 subunit J GN = EIF3J	4.3	1	0.783	0.007
Q01518	Adenylyl cyclase-associated protein 1 GN = CAP1	2.7	1	0.780	0.008
P10109	Adrenodoxin, mitochondrial GN = FDX1	4.9	1	0.772	0.044
P01717	Immunoglobulin lambda variable 3-25 GN = IGLV3-25	36.6	1	0.767	0.007
P01834	Immunoglobulin kappa constant GN = IGKC	79.4	1	0.765	0.027
Q9BQT9	Calsyntenin-3 GN = CLSTN3	6.2	5	0.750	0.047
P00966	Argininosuccinate synthase GN = ASS1	17.7	7	0.746	0.023
Q9NQ29	Putative RNA-binding protein Luc7-like 1 GN = LUC7L	4.9	1	0.745	0.028
P31939	Bifunctional purine biosynthesis protein PURH GN = ATIC	2.5	1	0.734	0.017
P0DOX8	Immunoglobulin lambda-1 light chain GN = NA	45.4	4	0.722	0.025
P0DOX5	Immunoglobulin gamma-1 heavy chain GN = NA	47.7	10	0.719	0.027
P84077	ADP-ribosylation factor 1 GN = ARF1	17.7	3	0.716	0.033
P38159	RNA-binding motif protein, X chromosome GN = RBMX	14.1	5	0.709	0.044
P06576	ATP synthase subunit beta, mitochondrial GN = ATP5F1B	4.5	2	0.696	0.024
Q6UXH9	Inactive serine protease PAMR1 GN = PAMR1	2.5	2	0.684	0.036
P05019	Insulin-like growth factor I GN = IGF1	13.8	3	0.671	0.038
P78563	Double-stranded RNA-specific editase 1 GN = ADARB1	1.2	1	0.652	0.003
A0A0J9YXX1	Immunoglobulin heavy variable 5-10-1 GN = IGHV5-10-1	32.5	2	0.648	0.007
Q92496	Complement factor H-related protein 4 GN = CFHR4	17.5	3	0.632	0.014
A0A0C4DH38	Immunoglobulin heavy variable 5-51 GN = IGHV5-51	53.0	3	0.631	0.024
A0A075B6K6	Immunoglobulin lambda variable 4-3 GN = IGLV4-3	8.2	1	0.552	0.017
Q15848	Adiponectin GN = ADIPOQ	12.3	2	0.476	0.027

**Table 2:** List of differentially expressed proteins identified in the follicular fluid from normal-weight patients with PCOS compared with the controls.

**Accession**	**Description**	**Coverage %**	**Unique peptides**	**Fold change**	***P*-value**
P02741	C-reactive protein GN = CRP	24.6	7	2.513	0.026
P51460	Insulin-like 3 GN = INSL3	32.1	4	1.494	0.033
Q9UGM5	Fetuin-B GN = FETUB	14.7	5	1.480	0.016
Q14520	Hyaluronan-binding protein 2 GN = HABP2	28.9	14	1.429	0.028
P18428	Lipopolysaccharide-binding protein GN = LBP	18.5	7	1.371	0.033
P51149	Ras-related protein Rab-7a GN = RAB7A	6.8	1	1.312	0.048
Q9NS84	Carbohydrate sulfotransferase 7 GN = CHST7	8.2	4	1.298	0.001
P04180	Phosphatidylcholine-sterol acyltransferase GN = LCAT	28.0	9	1.248	0.015
Q9NR30	Nucleolar RNA helicase 2 GN = DDX21	1.0	1	0.821	0.022
A0A0B4J1X8	Immunoglobulin heavy variable 3-43 GN = IGHV3-43	18.6	1	0.804	0.036
P48061	Stromal cell-derived factor 1 GN = CXCL12	39.8	4	0.799	0.041
P01834	Immunoglobulin kappa constant GN = IGKC	79.4	1	0.780	0.027
P52907	F-actin-capping protein subunit alpha-1 GN = CAPZA1	9.1	1	0.762	0.032
Q6UXH9	Inactive serine protease PAMR1 GN = PAMR1	2.5	2	0.748	0.024
P68871	Hemoglobin subunit beta GN = HBB	70.1	8	0.733	0.034
P16870	Carboxypeptidase E GN = CPE	6.1	3	0.711	0.049
Q96KG9	N-terminal kinase-like protein GN = SCYL1	2.6	2	0.695	0.032
P27482	Calmodulin-like protein 3 GN = CALML3	13.4	1	0.564	0.043
Q9UBG3	Cornulin OS = Homo sapiens GN = CRNN	2.0	1	0.286	0.018

### Functional Enrichment and Clustering Analyses of DEPs

**Figure 3:**
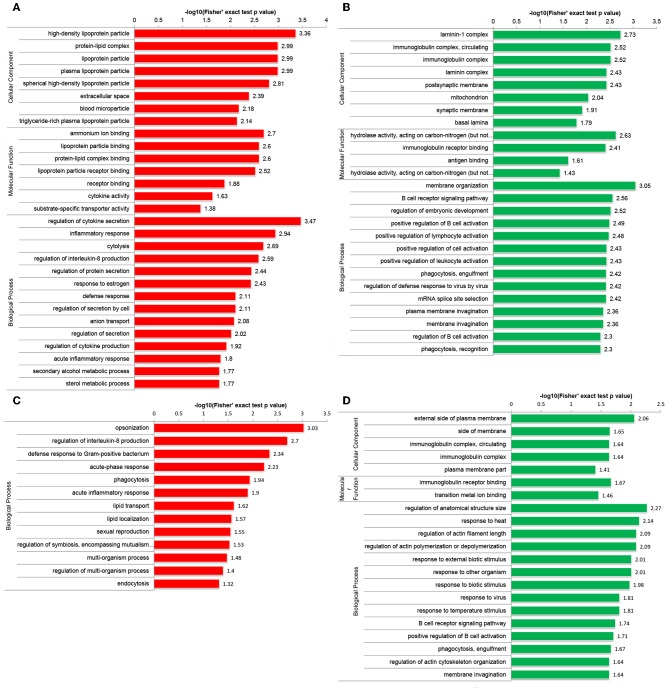
Gene Oncology (GO) enrichment analysis of differentially expressed proteins (DEPs) in follicular fluid of PCOS women and controls. Upregulated DEPs **(A)** and downregulated DEPs **(B)** in GO functional enrichment analysis of Po vs. Control. Upregulated DEPs **(C)** and downregulated DEPs **(D)** in GO functional enrichment analysis of Pn vs. Control.

Functional enrichment analyses were performed to investigate the biological roles of DEPs. Interestingly, GO enrichment analysis demonstrated that DEPs were co-enriched in several inflammatory and immune-related biological processes in both the Po and Pn groups. For example, upregulated DEPs were co-enriched in the regulation of interleukin-8 (IL-8) production and acute inflammatory response (Figures 3A,C). The downregulated DEPs were associated with cellular component of circulating immunoglobulin complex. They were also enriched in the processes of B cell receptor signaling pathway, positive regulation of B cell activation and phagocytosis, engulfment in both Po and Pn (Figures 3B,D). The results indicated that PCOS was closely related to the alterations in inflammatory and immunological environment of FF. Moreover, downregulated proteins in Po were found to be associated with several terms including cellular component of laminin-1 complex, laminin complex, basal lamina, and hydrolase activity.

**Figure 4:**
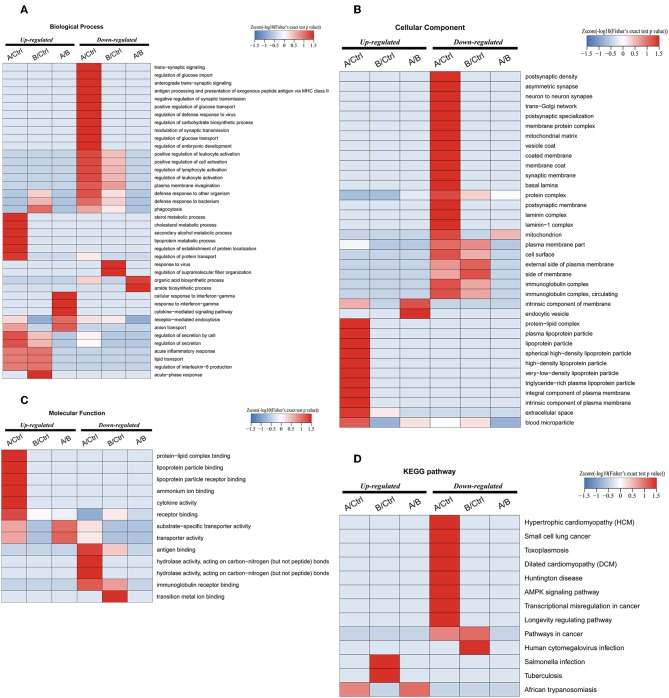
Functional enrichment-based clustering analyses of differentially expressed proteins in follicular fluid of PCOS patients and controls. **(A)** Biological process analysis. **(B)** Cellular component analysis. **(C)** Molecular function analysis. **(D)** KEGG pathway analysis.

**Figure 5:**
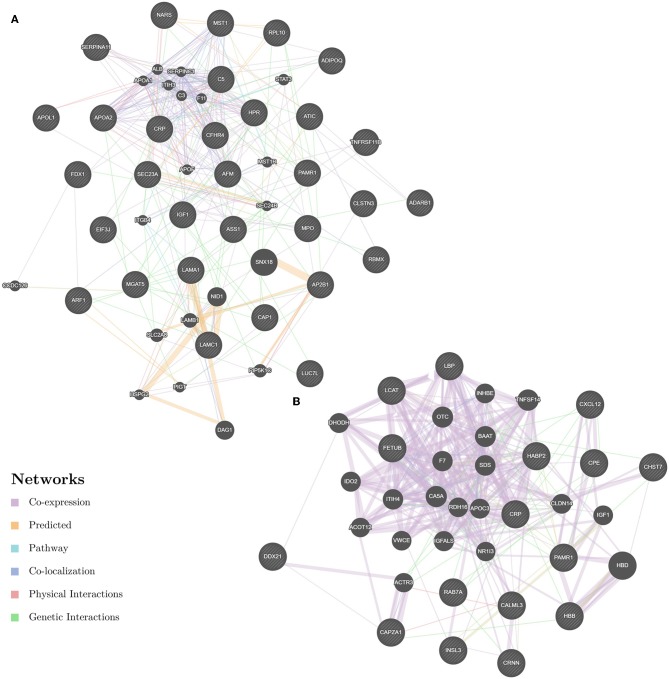
GeneMANIA network revealing potential protein interactions of differentially expressed proteins and associated proteins inferred from the literature. **(A)** GeneMANIA network of Po vs. Control. **(B)** GeneMANIA network of Pn vs. Control.

Enrichment-based clustering analyses were carried out to identify features of the DEPs (Figure 4). In biological process category, upregulated DEPs in Po/C were mainly enriched in metabolic-related processes such as sterol metabolic, cholesterol metabolic, secondary alcohol metabolic and lipoprotein metabolic processes (Figure 4A). Regarding cellular component and molecular function categories, the upregulated DEPs in Po/C were associated with terms including protein-lipid complex, lipoprotein particle, protein-lipid complex binding, lipoprotein particle binding, and lipoprotein particle receptor binding (Figures 4B,C). The KEGG pathway-based enrichment analysis showed that the downregulated proteins were enriched in the AMPK signaling pathway of the Po/C (Figure 4D). GeneMANIA is flexible in generating potential interactions using a wealth of genomics and proteomic data (21). We performed the GeneMANIA network to illustrate potential patterns of co-expression, co-localization, pathways, and interactions involved in PCOS (Figure 5).

### Gene Set Enrichment Analysis

**Figure 6:**
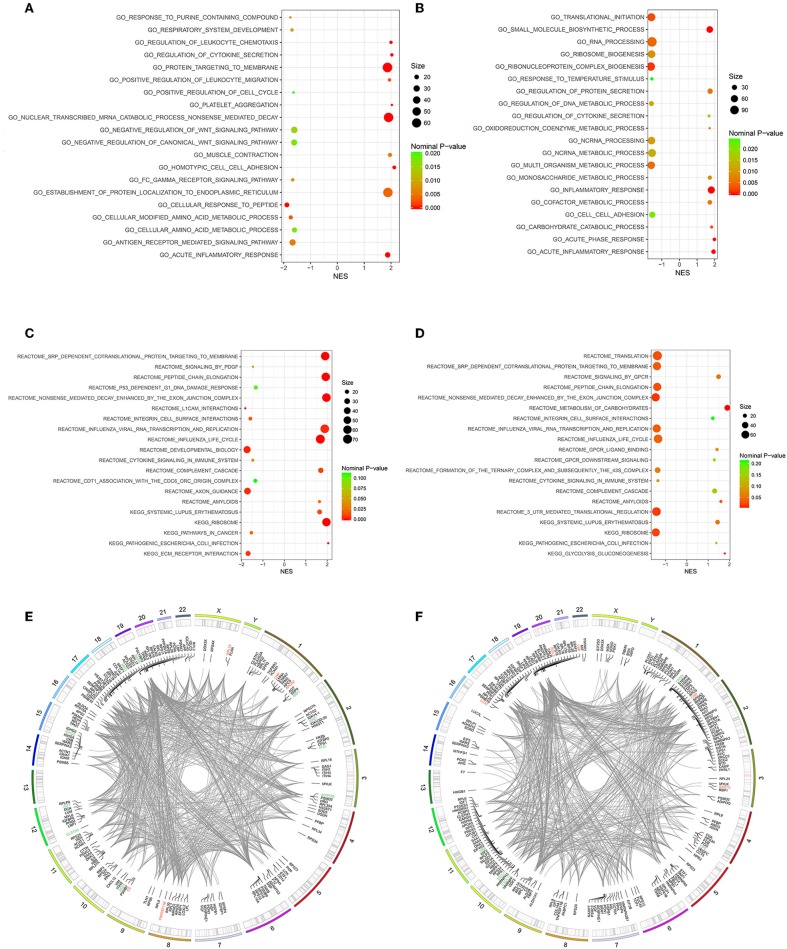
Gene Set Enrichment Analysis (GSEA)-Gene Ontology enrichment of follicular fluid from PCOS patients and controls. **(A)** Top10 upregulated and downregulated biological processes of Po vs. Control. **(B)** Top 10 upregulated and downregulated biological processes of Pn vs. Control. **(C)** Top10 upregulated and downregulated KEGG or Reactome pathways of Po vs. Control. **(D)** Top10 upregulated and downregulated KEGG or Reactome pathways of Pn vs. Control. **(E,F)** RCircos and GSEA-STRING analysis indicating chromosomal localization, heatmaps of protein expression, and interactions of proteins within the leading edge gene sets of top enriched biological processes in Po vs. Control and Pn vs. Control, respectively. Upregulated DEPs were shown in red color, and the downregulated DEPs were shown in green.

Owing to the limited number of DEPs examined, we conducted the GSEA analysis using a pre-defined collection of gene sets to further explore our findings. The positive value of NES showed an increment, while the negative value of NES indicated a reduction. Several biological processes were identified in accordance with our function enrichment analysis of DEPs. Acute inflammatory response and the regulation of cytokine secretion processes were co-enriched in both the Po and Pn groups according to the GSEA GO enrichment analysis (Figures 6A,B). In addition, homotypic cell-cell adhesion, regulation of leukocyte chemotaxis, and platelet aggregation were significantly altered in the Po group. Metabolic-related processes were also enriched in the Pn group, including the carbohydrate catabolic, cofactor metabolic, and monosaccharide metabolic processes. Enriched pathways were identified by Reactome and KEGG pathways based on the GSEA analysis (Figures 6C,D). The complement cascade pathway was enriched in both Po and Pn groups. Furthermore, significantly altered L1CAM interactions, extracellular matrix receptor interaction, and integrin cell surface interactions were found to be associated with PCOS. All overlapping genes involved in each of the top 10 enriched biological processes were retrieved by leading-edge analysis and visualized by the RCircos package, suggesting a wide range of interactions among these core enrichment genes (Figures 6E,F).

### Validation of DEPs by ELISA Analysis

**Figure 7:**
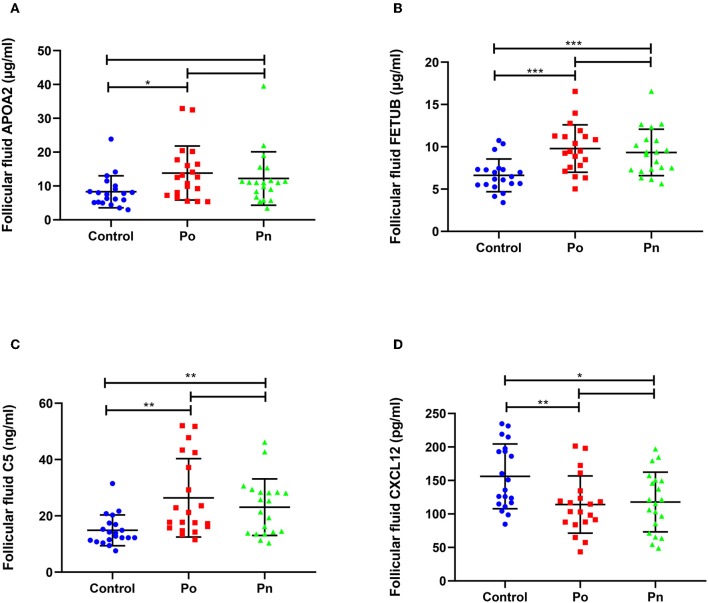
Validation of differentially expressed proteins in follicular fluid of overweight/obese PCOS patients, normal-weight PCOS patients and the controls. Graphical results are shown in mean ± SD, *P < 0.05, **P < 0.01, ***P < 0.001. **(A)** Apolipoprotein A-II (APOA2). **(B)** Fetuin-B (FETUB). **(C)** Complement C5 (C5). **(D)** Stromal cell-derived factor 1 (CXCL12).

We selected four DEPs involved in different biological processes, that could be validated by commercial ELISA kits using FF samples taken from 20 patients from each of the Po, Pn, and control groups (Figure 7). Consistent with the proteomic analysis, the level of APOA2 was significantly higher in the Po group than that in the control group (Figure 7A). In addition, significantly higher levels of FETUB and C5 were found in both Po/C and Pn/C (Figures 7B,C). Moreover, FF levels of CXCL12 in the Po and Pn groups were significantly lower than that in the control group (Figure 7D). Subgroup analyses of ELISA validation results were also conducted (Supplementary Figures 1, 2).

### Clinical, Hormonal, and Biochemical Characteristics of Overweight/Obese and Normal-Weight PCOS Patients

We compared the clinical, hormonal, and biochemical features of 75 overweight/obese PCOS and 48 normal-weight PCOS patients. As shown in Table 3, significantly higher levels of testosterone, fasting insulin, HOMA-IR, fasting glucose, aspartate aminotransferase (AST), alanine aminotransferase (ALT), gamma-glutamyltransferase (GGT), triglyceride, apolipoprotein B (apoB), and lower levels of luteinizing hormone (LH), ratio of LH/FSH, AMH, HDL-C, and apolipoprotein A1 (apoA1) were identified in the Po group than those in the Pn group. Furthermore, the Po group presented a significantly lower mean number of retrieved oocytes (16.77 ± 6.84 vs. 20.69 ± 8.65, *P* = 0.01) and cleavage embryos (11.60 ± 6.11 vs. 14.50 ± 8.01, *P* = 0.035) on IVF. The results indicate that obesity could aggravate hormone imbalances, metabolic disturbances, and IVF outcomes in PCOS patients. In addition, we conducted the subgroup analyses to further detect the impact of obesity and hyperandrogenism on PCOS (Supplementary Tables 1, 2).

**Table 3:** Clinical, hormonal, and biochemical characteristics of overweight/obese and normal-weight patients with PCOS.

	**PCOS (BMI ≥ 25 kg/m^**2**^) *N* = 75**	**PCOS (BMI < 25 kg/m^**2**^) *N* = 48**	***P-*value**
Age (year)	29.56 ± 3.10	30.52 ± 2.95	0.090
BMI (kg/m^2^)	28.90 ± 2.95	21.87 ± 3.47	0.000
Testosterone (ng/ml)	0.77 ± 0.34	0.65 ± 0.17	0.008
Estradiol (pg/ml)	65.69 ± 63.57	70.35 ± 44.07	0.658
LH (mIU/ml)	10.23 ± 5.60	12.92 ± 6.15	0.013
FSH (mIU/ml)	6.65 ± 2.04	7.01 ± 2.01	0.332
LH/FSH ratio	1.58 ± 0.80	1.90 ± 0.92	0.049
Prolactin (ng/ml)	11.82 ± 12.01	10.15 ± 5.32	0.366
Progesterone (ng/ml)	0.73 ± 1.26	1.27 ± 3.54	0.316
AMH (ng/ml)	7.66 ± 4.24	9.60 ± 4.95	0.022
AST (U/L)	22.96 ± 10.34	16.79 ± 5.89	0.000
ALT (U/L)	32.16 ± 22.12	18.56 ± 12.53	0.000
GGT (U/L)	30.69 ± 18.11	19.00 ± 11.13	0.000
ALP (U/L)	77.75 ± 16.99	73.23 ± 16.04	0.143
Total cholesterol (mmol/L)	4.89 ± 0.87	4.76 ± 0.81	0.392
Triglyceride (mmol/L)	1.87 ± 1.18	1.27 ± 0.70	0.002
HDL-C (mmol/L)	1.07 ± 0.27	1.41 ± 0.36	0.000
LDL-C (mmol/L)	3.12 ± 0.74	2.88 ± 0.66	0.069
ApoA1 (g/L)	1.38 ± 0.21	1.60 ± 0.27	0.000
ApoB (g/L)	0.95 ± 0.24	0.81 ± 0.18	0.001
Fasting insulin (mU/L)	19.52 ± 7.98	12.58 ± 6.63	0.000
Fasting glucose (mmol/L)	5.33 ± 0.61	5.01 ± 0.51	0.003
HOMA-IR	4.68 ± 2.11	2.87 ± 1.84	0.000
No. of oocytes retrieved	16.77 ± 6.84	20.69 ± 8.65	0.010
No. of cleavage embryos	11.60 ± 6.11	14.50 ± 8.01	0.035
No. of good-quality embryos on day 3	5.55 ± 5.24	6.10 ± 4.94	0.557

*Data was presented as mean ± standard deviation (SD). BMI, body mass index; LH, luteinizing hormone; FSH, follicle-stimulating hormone; AMH, anti-Müllerian hormone; AST, aspartate aminotransferase; ALT, alanine aminotransferase; GGT, gamma-glutamyltransferase; ALP, alkaline phosphatase; HDL-C, high density lipoprotein cholesterol; LDL-C, low density lipoprotein cholesterol; ApoA1, apolipoprotein A1; ApoB, apolipoprotein B*.

## Discussion

In the present study, we used high-throughput proteomic techniques to investigate the protein profiles of FF samples in overweight/obese PCOS patients, normal-weight PCOS patients, and non-PCOS women. Among the DEPs, 47 proteins were found to be linked to PCOS for the first time. Through the GO and GSEA analyses, inflammatory, immunological, and metabolic alterations were identified in the follicular environment of PCOS. Moreover, obesity could worsen disturbances by analyses of clinical features and proteomic data.

CRP, PAMR1, and IGKC were identified in the FF of both Po/C and Pn/C. CRP has been identified as an inflammatory marker. Higher levels of CRP indicate that PCOS patients are accompanied by a low-grade chronic inflammation (22). In addition, higher CRP levels in PCOS patients were found to be linked with heritability (23). Elevated circulating levels of CRP have been observed in several diseases related to IR, such as type 2 diabetes mellitus (T2DM), atherosclerosis, cardiovascular disease, and metabolic syndrome (24). Furthermore, CRP levels were found positively associated with the adipose-IR index in PCOS patients, indicating that chronic inflammation may induce IR in the adipose tissue (25). For the first time, we observed the downregulation of PAMR1 and IGKC in PCOS patients, suggesting their potential roles in follicular development. Peptidase domain-containing protein associated with muscle regeneration 1 (PAMR1) was first found to be involved in the regeneration of skeletal muscle (26). It has also been reported as an epigenetically inactivated gene in breast cancer tissues, involving in the cell growth (27). Coronary artery disease and T2DM are known complications of PCOS. PAMR1 has been found associated with the coronary artery disease in adipose tissue (28). Moreover, it has been reported as an endoplasmic reticulum-stress-induced protein related to islet function during T2DM (29). IGKC, the C-terminal constant region of immunoglobulin kappa light chain, has been identified as an immunologic biomarker related to plasma cells (30). It is associated with prognosis and response to chemotherapy in several tumors, including breast cancer, non-small cell lung cancer, and colorectal cancer (31). Differentially expression of IGKC found in our study may extend the current knowledge of plasma cell infiltration and immune response in the process of PCOS.

PCOS is recognized as a state of chronic low-grade inflammation linked to several autoimmune diseases. In the current study, upregulated proteins in PCOS were enriched in inflammatory and immunological processes, such as the regulation of IL-8 production. IL-8 was found to activate vascular endothelial growth factor receptor pathways and to regulate the steroidogenesis of granulosa cells (32, 33). Significantly higher levels of C5 were identified and validated from FF samples of PCOS patients in this study. Anaphylatoxin C5a induced inflammatory responses and reduced insulin sensitivity by activating TLR4/NF-kB/PI3 K signaling pathway (34). Similarly, several acute-phase response proteins were identified in the plasma from PCOS (35). In addition, both lean and obese PCOS patients have higher levels of inflammatory markers compared to the controls (36). Elevated transcripts encoding cytokines, chemokines as well as immune cell markers in granulosa cells, unbalanced ovarian recruitment of macrophages, decreased dendritic cells, altered T lymphocyte subsets, and leukocyte populations have been reported in PCOS patients and animal models, implying immunological and inflammatory imbalance of the disease (37–39). Obesity increased the expression of early complement pathway components in subcutaneous adipose tissue (40). Obesity has been reported to upregulate inflammatory adipokines and to induce IR, androgen production, and adipogenesis (41, 42). Hence, pro-inflammatory follicular environment in PCOS could be amplified by obesity.

In the present study, several metabolic-related proteins were found to be differentially expressed in PCOS. A group of studies showed higher metabolic-related risks of IR, T2D, metabolic syndrome, and cardiovascular disease in PCOS (43, 44). Compared to the Pn group, we found a higher prevalence of metabolic disturbance, lower numbers of retrieved oocytes and cleavage embryos in IVF in the Po group. APOA2 has been reported to be linked to obesity and IR (45, 46). We found significantly higher levels of APOA2 in FF from the Po group than that of the control group, indicating the effect of excessive weight on metabolism. Obesity is associated to worse metabolic complications and reproductive outcomes in PCOS patients and also to higher hyperandrogenism, IR, hyperinsulinemia, and adipokines. Obesity could amplify hyperandrogenism in PCOS, resulting in increased total testosterone, free androgen index, hirsutism, and decreased hormone-binding globulin (14). Increased incidence of metabolic syndrome was found in overweight/obese patients but not in lean patients (43). However, non-obese PCOS women still have increased incidence of metabolic disturbances and long-term metabolic complications as reported according to a recent systematic review and meta-analysis (47). A serum proteomics study of normal-weight PCOS adolescents showed the enrichment of inflammatory and immune responses, metabolism, and insulin-like growth factor receptor signaling pathway (48). Our findings suggest that the metabolic disturbance might not only be a consequent of obesity, but also an intrinsic etiologic basis for PCOS.

Several DEPs found in our study have also been reported in previous studies. Follicular basal lamina is involved in the developmental competence of oocytes, follicular development, and ovulation (49–51). We found the downregulation of laminin subunit gamma-1 and laminin subunit alpha-1 in PCOS. Similarly, collagens, laminins, and heparin sulfate proteoglycan 2 have been reported to be downregulated in PCOS (20). Moreover, downregulated genes of extracellular matrix and cell adhesion molecules were found in the cumulus cells of PCOS patients (52). These results highlighted the alterations of basal lamina matrix composition as a new perspective for understanding the etiology of PCOS. ADP-ribosylation factor 1 and protein transport protein Sec23A were elevated in the adipose tissue of PCOS women after aerobic exercise, indicating the roles of coatomer GTPases in lipolysis and triglyceride storage in the adipose tissue of PCOS (53). Lower levels of adiponectin in PCOS patients have been reported by earlier studies and may be regarded as novel biomarkers of PCOS (54, 55). Insulin-like 3 (INSL3) is produced by theca interna cells of growing antral follicles, involved in androstenedione synthesis, estradiol production, follicular growth, and ovulation (56, 57). A significant negative correlation between BMI and INSL3, as well as a positive correlation between INSL3 and testosterone were found in men (58). INSL3 and AMH levels are increased in patients of amenorrhea and oligomenorrhea, reflecting a dysfunction of the thecal and granulosa cells in PCOS (59).

The limitations of the study included patient heterogeneity, relatively small sample size, and cross-sectional study design. For more authentic results, further studies are required on extended population and different sample cohorts to supplement our findings. Moreover, it remains unclear whether these proteins contribute directly to the pathogenesis of PCOS and need to be clarified in the future.

In summary, this is the first study to quantitatively profile the FF proteome in overweight/obese PCOS patients, normal-weight PCOS patients, and non-PCOS women. A panel of proteins associated with inflammatory, immunological, and metabolic alterations were found in the FF of PCOS patients, which could be aggravated by obesity. Moreover, APOA2, C5, FETUB, and CXCL12 were identified as differentially expressed and validated. The results provided us with a better knowledge of the intra-follicular abnormalities in PCOS. Further prospective studies are needed to clarify the roles of these proteins in the molecular mechanisms underlying PCOS.

## Data Availability Statement

The mass spectrometry proteomics data have been deposited to the ProteomeXchange Consortium (http://proteomecentral.proteomexchange.org) by the PRIDE partner repository with the dataset identifier PXD013937.

## Ethics Statement

The studies involving human participants were reviewed and approved by Ethics Committee at Shengjing Hospital of China Medical University. The patients/participants provided their written informed consent to participate in this study. Written informed consent was obtained from the individual(s) for the publication of any potentially identifiable images or data included in this article.

## Author Contributions

XZ: study design, bioinformatics, and ELISA analysis. XX, LK, and JQ: sample preparation and proteomic analysis. PL: ELISA analysis and data analysis. FZ: sample preparation and critical discussion. ZY: critical discussion and manuscript revision. JT: study design, critical discussion, and manuscript revision. All of the authors contributed to the manuscript writing.

### Conflict of Interest

The authors declare that the research was conducted in the absence of any commercial or financial relationships that could be construed as a potential conflict of interest.
