# Supplementary material for: TMT Based Proteomic Analysis of Human Follicular Fluid From Overweight/Obese and Normal-Weight Patients With Polycystic Ovary Syndrome
Source: Front Endocrinol (Lausanne). 2019 Nov 26;10:821. doi: 10.3389/fendo.2019.00821 (PMC6966116; doi:10.3389/fendo.2019.00821)
Supplement: Supplementary file 1 [file Data_Sheet_1.docx]

Supplementary Material

## 1 Supplementary Figures


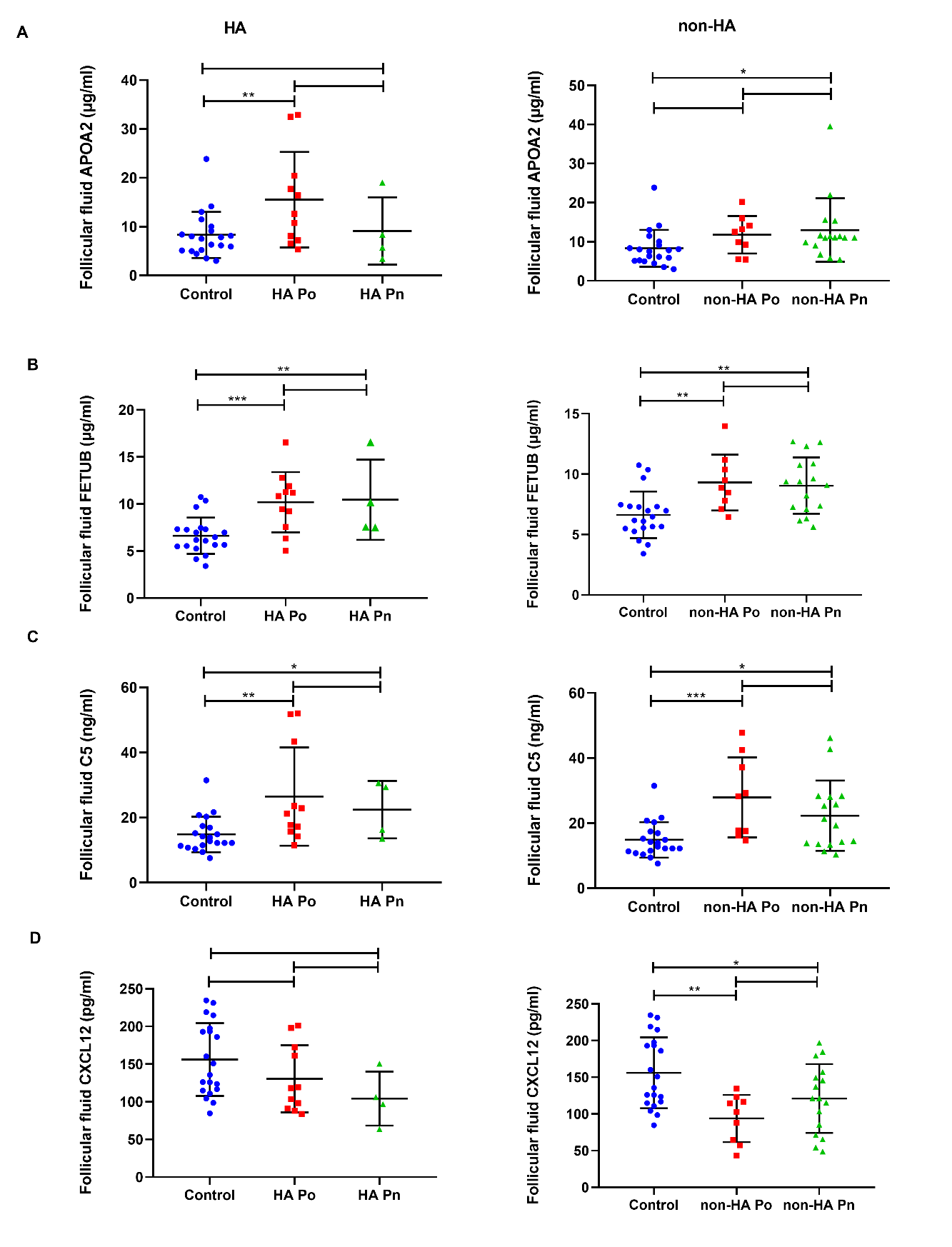


**Supplementary Figure 1.** Subgroup analyses of differentially expressed proteins in follicular fluid of PCOS patients and controls. The graphical results were displayed in mean ± SD, **P* < 0.05, ***P* < 0.01, ****P* < 0.001. (A) Apolipoprotein A-II (APOA2). (B) Fetuin-B (FETUB). (C) Complement C5 (C5). (D) stromal cell-derived factor 1 (CXCL12). HA: hyperandrogenism; Po: overweight/obese PCOS patients; Pn: normal-weight PCOS patients.


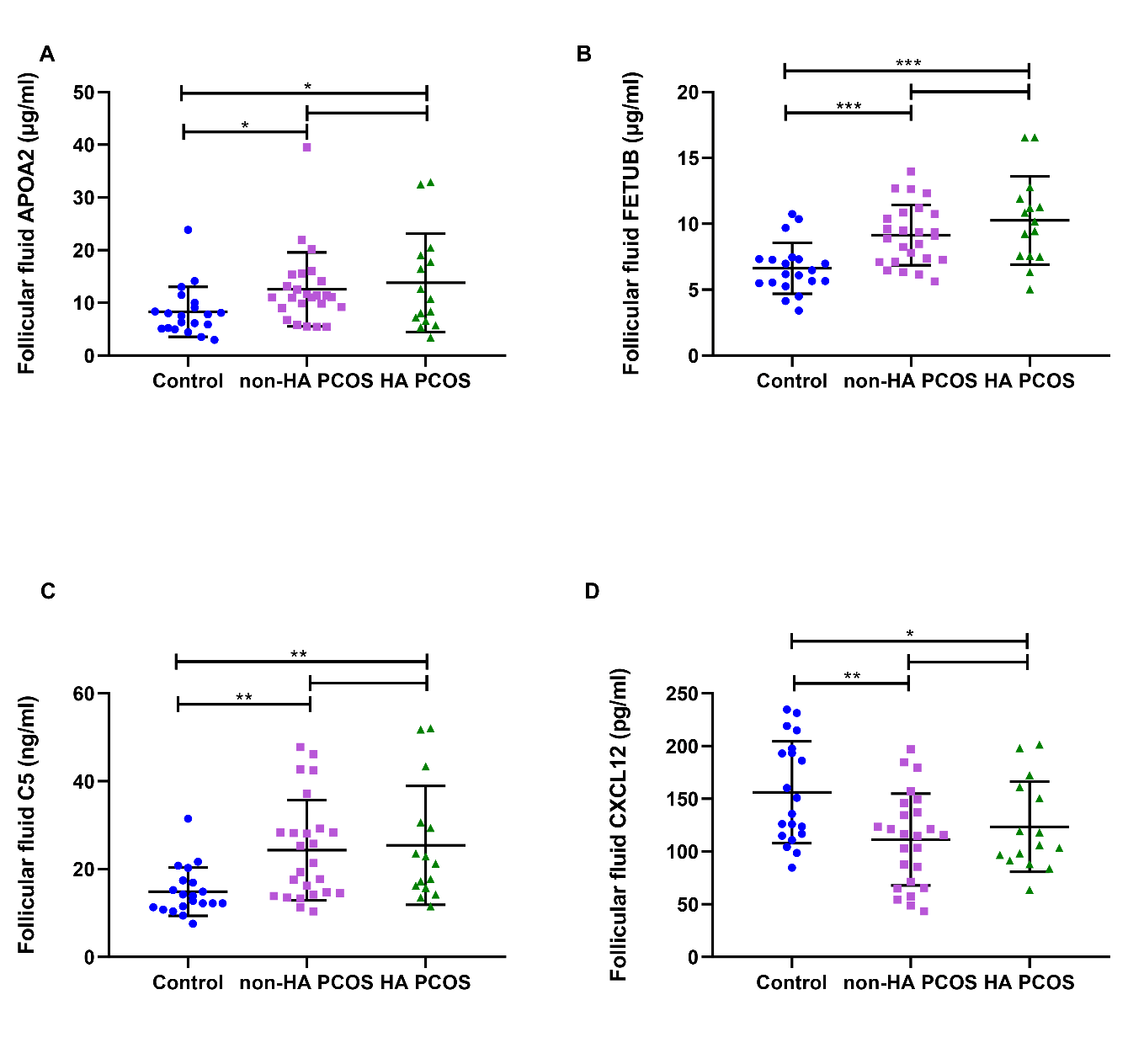


**Supplementary Figure 2.** Validation of differentially expressed proteins in follicular fluid of hyperandrogenism (HA) PCOS patients, non-hyperandrogenism (non-HA) PCOS patients and the controls. The graphical results were displayed in mean ± SD, **P* < 0.05, ***P* < 0.01, ****P* < 0.001. (A) Apolipoprotein A-II (APOA2). (B) Fetuin-B (FETUB). (C) Complement C5 (C5). (D) stromal cell-derived factor 1 (CXCL12).

**Supplementary Table 1**

| **Clinical, hormonal, and biochemical characteristics of overweight/obese and normal-weight patients with PCOS** | | | | | | |
| --- | --- | --- | --- | --- | --- | --- |
|  | **PCOS (BMI≥25 kg/m^2^）** | | | **PCOS (BMI＜25 kg/m^2^）** | | |
|  | **Hyperandrogenism N = 31** | **Non-hyperandrogenism N = 44** | ***P*-value** | **Hyperandrogenism N = 7** | **Non-hyperandrogenism N = 41** | ***P*-value** |
| Age (year) | 29.12 ± 3.48 | 29.93 ± 2.78 | 0.257 | 29.50 ± 3.51 | 30.56 ± 2.83 | 0.409 |
| Testosterone (ng/ml) | 1.06 ± 0.37 | 0.58 ± 0.13 | <0.001 | 0.95 ± 0.11 | 0.60 ± 0.13 | <0.001 |
| Estradiol (pg/ml) | 88.49 ± 95.32 | 56.93 ± 34.69 | 0.043 | 122.50 ± 58.45 | 62.90 ± 37.27 | 0.001 |
| LH (mIU/ml) | 11.97 ± 6.86 | 9.17 ± 5.18 | 0.043 | 17.63 ± 5.69 | 12.17 ± 6.03 | 0.043 |
| FSH (mIU/ml) | 5.95 ± 2.08 | 6.94 ± 2.05 | 0.041 | 6.60 ± 0.88 | 7.03 ± 2.13 | 0.629 |
| LH/FSH ratio | 2.05 ± 1.18 | 1.37 ± 0.71 | 0.002 | 2.68 ± 0.89 | 1.78 ± 0.88 | 0.024 |
| Prolactin (ng/ml) | 17.74 ± 25.13 | 10.90 ± 6.80 | 0.083 | 8.80 ± 1.07 | 10.41 ± 5.71 | 0.499 |
| Progesterone (ng/ml) | 0.87 ± 1.29 | 0.81 ± 1.66 | 0.863 | 4.01 ± 8.58 | 0.90 ± 2.06 | 0.045 |
| AMH (ng/ml) | 7.67 ± 4.32 | 7.83 ± 4.28 | 0.874 | 8.54 ± 5.29 | 9.69 ± 4.99 | 0.605 |
| AST (U/L) | 24.25 ± 12.09 | 21.63 ± 8.67 | 0.268 | 14.50 ± 3.62 | 17.10 ± 6.18 | 0.323 |
| ALT (U/L) | 36.34 ± 25.87 | 28.37 ± 18.23 | 0.114 | 11.00 ± 3.52 | 19.85 ± 13.08 | 0.109 |
| GGT (U/L) | 31.34 ± 17.60 | 29.72 ± 18.26 | 0.696 | 15.17 ± 3.71 | 19.51 ± 11.88 | 0.382 |
| ALP (U/L) | 76.90 ± 17.50 | 77.23 ± 17.44 | 0.935 | 76.83 ± 14.35 | 73.00 ± 16.45 | 0.592 |
| Total cholesterol (mmol/L) | 4.95 ± 0.87 | 4.83 ± 0.88 | 0.568 | 4.18 ± 1.20 | 4.82 ± 0.72 | 0.068 |
| Triglyceride (mmol/L) | 1.73 ± 0.78 | 1.96 ± 1.36 | 0.406 | 0.81 ± 0.23 | 1.36 ± 0.72 | 0.077 |
| HDL-C (mmol/L) | 1.12 ± 0.27 | 1.04 ± 0.26 | 0.192 | 1.48 ± 0.20 | 1.37 ± 0.36 | 0.483 |
| LDL-C (mmol/L) | 3.17 ± 0.72 | 3.07 ± 0.77 | 0.537 | 2.82 ± 0.47 | 2.89 ± 0.69 | 0.812 |
| ApoA1 (g/L) | 1.41 ± 0.19 | 1.35 ± 0.21 | 0.186 | 1.59 ± 0.17 | 1.59 ± 0.27 | 0.973 |
| ApoB (g/L) | 0.99 ± 0.23 | 0.92 ± 0.25 | 0.236 | 0.75 ± 0.11 | 0.82 ± 0.19 | 0.370 |
| Fasting insulin (mU/L) | 21.20 ± 8.61 | 18.57 ± 8.03 | 0.171 | 13.22 ± 13.98 | 12.55 ± 5.20 | 0.823 |
| Fasting glucose (mmol/L) | 5.40 ± 0.60 | 5.33 ± 0.63 | 0.597 | 5.20 ± 0.81 | 4.98 ± 0.47 | 0.334 |
| HOMA-IR | 5.12 ± 2.22 | 4.47 ± 2.20 | 0.211 | 3.44 ± 4.41 | 2.80 ± 1.22 | 0.442 |
| No. of oocytes retrieved | 17.06 ± 7.18 | 17.93 ± 8.31 | 0.632 | 17.33 ± 4.68 | 20.71 ± 8.61 | 0.356 |
| No. of cleavage embryos | 11.34 ± 5.27 | 12.65 ± 7.39 | 0.392 | 12.00 ± 4.98 | 14.29 ± 7.58 | 0.479 |
| No. of good-quality embryos on day 3 | 6.03 ± 4.75 | 5.67 ± 5.75 | 0.773 | 4.67 ± 4.89 | 6.15 ± 4.92 | 0.494 |
| Data was presented as mean ± standard deviation (SD). | | | | | | |

**Supplementary Table 2**

| **Clinical, hormonal, and biochemical characteristics of Hyperandrogenism and Non-hyperandrogenism patients with PCOS** | | | | | | |
| --- | --- | --- | --- | --- | --- | --- |
|  | **Hyperandrogenism** | | | **Non-hyperandrogenism** | | |
|  | **PCOS (BMI≥25 kg/m^2^） N = 31** | **PCOS (BMI＜25 kg/m^2^） N = 7** | ***P*-value** | **PCOS (BMI≥25 kg/m^2^） N = 44** | **PCOS (BMI＜25 kg/m^2^） N = 41** | ***P*-value** |
| Age (year) | 29.12 ± 3.48 | 29.50 ± 3.51 | 0.810 | 29.93 ± 2.78 | 30.56 ± 2.83 | 0.301 |
| BMI (kg/m2) | 29.38 ± 2.70 | 21.72 ± 1.95 | <0.001 | 28.32 ± 3.12 | 21.97 ± 3.66 | <0.001 |
| Testosterone (ng/ml) | 1.06 ± 0.37 | 0.95 ± 0.11 | 0.491 | 0.58 ± 0.13 | 0.60 ± 0.13 | 0.608 |
| Estradiol (pg/ml) | 88.49 ± 95.32 | 122.50 ± 58.45 | 0.407 | 56.93 ± 34.69 | 62.90 ± 37.27 | 0.442 |
| LH (mIU/ml) | 11.97 ± 6.86 | 17.63 ± 5.69 | 0.066 | 9.17 ± 5.18 | 12.17 ± 6.03 | 0.014 |
| FSH (mIU/ml) | 5.95 ± 2.08 | 6.60 ± 0.88 | 0.461 | 6.94 ± 2.05 | 7.03 ± 2.13 | 0.834 |
| LH/FSH ratio | 2.05 ± 1.18 | 2.68 ± 0.89 | 0.219 | 1.37 ± 0.71 | 1.78 ± 0.88 | 0.018 |
| Prolactin (ng/ml) | 17.74 ± 25.13 | 8.80 ± 1.07 | 0.395 | 10.90 ± 6.80 | 10.41 ± 5.71 | 0.717 |
| Progesterone (ng/ml) | 0.87 ± 1.29 | 4.01 ± 8.58 | 0.046 | 0.81 ± 1.66 | 0.90 ± 2.06 | 0.818 |
| AMH (ng/ml) | 7.67 ± 4.32 | 8.54 ± 5.29 | 0.665 | 7.83 ± 4.28 | 9.69 ± 4.99 | 0.066 |
| AST (U/L) | 24.25 ± 12.09 | 14.50 ± 3.62 | 0.060 | 21.63 ± 8.67 | 17.10 ± 6.18 | 0.007 |
| ALT (U/L) | 36.34 ± 25.87 | 11.00 ± 3.52 | 0.023 | 28.37 ± 18.23 | 19.85 ± 13.08 | 0.015 |
| GGT (U/L) | 31.34 ± 17.60 | 15.17 ± 3.71 | 0.033 | 29.72 ± 18.26 | 19.51 ± 11.88 | 0.003 |
| ALP (U/L) | 76.90 ± 17.50 | 76.83 ± 14.35 | 0.993 | 77.23 ± 17.44 | 73.00 ± 16.45 | 0.250 |
| Total cholesterol (mmol/L) | 4.95 ± 0.87 | 4.18 ± 1.20 | 0.070 | 4.83 ± 0.88 | 4.82 ± 0.72 | 0.973 |
| Triglyceride (mmol/L) | 1.73 ± 0.78 | 0.81 ± 0.23 | 0.007 | 1.96 ± 1.36 | 1.36 ± 0.72 | 0.013 |
| HDL-C (mmol/L) | 1.12 ± 0.27 | 1.48 ± 0.20 | 0.003 | 1.04 ± 0.26 | 1.37 ± 0.36 | <0.001 |
| LDL-C (mmol/L) | 3.17 ± 0.72 | 2.82 ± 0.47 | 0.260 | 3.07 ± 0.77 | 2.89 ± 0.69 | 0.268 |
| ApoA1 (g/L) | 1.41 ± 0.19 | 1.59 ± 0.17 | 0.041 | 1.35 ± 0.21 | 1.59 ± 0.27 | <0.001 |
| ApoB (g/L) | 0.99 ± 0.23 | 0.75 ± 0.11 | 0.021 | 0.92 ± 0.25 | 0.82 ± 0.19 | 0.056 |
| Fasting insulin (mU/L) | 21.20 ± 8.61 | 13.22 ± 13.98 | 0.068 | 18.57 ± 8.03 | 12.55 ± 5.20 | <0.001 |
| Fasting glucose (mmol/L) | 5.40 ± 0.60 | 5.20 ± 0.81 | 0.483 | 5.33 ± 0.63 | 4.98 ± 0.47 | 0.005 |
| HOMA-IR | 5.12 ± 2.22 | 3.44 ± 4.41 | 0.161 | 4.47 ± 2.20 | 2.80 ± 1.22 | <0.001 |
| No. of oocytes retrieved | 17.06 ± 7.18 | 17.33 ± 4.68 | 0.930 | 17.93 ± 8.31 | 20.71 ± 8.61 | 0.130 |
| No. of cleavage embryos | 11.34 ± 5.27 | 12.00 ± 4.98 | 0.780 | 12.65 ± 7.39 | 14.29 ± 7.58 | 0.310 |
| No. of good-quality embryos on day 3 | 6.03 ± 4.75 | 4.67 ± 4.89 | 0.524 | 5.67 ± 5.75 | 6.15 ± 4.92 | 0.683 |
| Data was presented as mean ± standard deviation (SD). | | | | | | |
